# Budd-Chiari Syndrome in a Patient with Hepatitis C

**DOI:** 10.1155/2016/7493970

**Published:** 2016-07-20

**Authors:** Joseph Frankl, Charles Hennemeyer, Michael S. Flores, Archita P. Desai

**Affiliations:** ^1^University of Arizona College of Medicine, Tucson, AZ 85724, USA; ^2^Department of Medical Imaging, University of Arizona College of Medicine, Tucson, AZ 85724, USA; ^3^Department of Pathology, University of Arizona College of Medicine, Tucson, AZ 85724, USA; ^4^Division of Gastroenterology, Department of Medicine, University of Arizona College of Medicine, Tucson, AZ 85724, USA

## Abstract

Chronic Budd-Chiari syndrome can present with cirrhosis and signs and symptoms similar to those of other chronic liver diseases. We present a case of Budd-Chiari syndrome discovered during attempted transjugular intrahepatic portosystemic shunting in a patient with decompensated cirrhosis believed to be secondary to hepatitis C. Although the patient had hepatocellular carcinoma, the Budd-Chiari syndrome was a primary disease due to hepatic venous webs. Angioplasty was performed in this case, which resolved the patient's symptoms related to portal hypertension. Follow-up venography 5 months after angioplasty demonstrated continued patency of the hepatic veins. A biopsy was obtained in the same setting, which showed centrilobular fibrosis indicating that venous occlusion was indeed the cause of cirrhosis. It is important to consider a second disease when treating a patient with difficult to manage portal hypertension.

## 1. Introduction

Budd-Chiari syndrome (BCS) is a rare disorder characterized by hepatic venous outflow tract obstruction at any level from the small hepatic veins to the terminal inferior vena cava (IVC) [1]. It progressively leads to sinusoidal congestion, hypoxic damage to the adjacent parenchyma, progressive centrilobular fibrosis, and ultimately cirrhosis [2]. BCS is usually considered after common causes of liver disease have been excluded due to its rarity. Here, we report a case of BCS in a patient with cirrhosis believed to be secondary to hepatitis C that was not detected until attempting transjugular intrahepatic portosystemic shunting (TIPS).

## 2. Case Presentation

Institutional review board approval was not required for this case report. A 67-year-old male with a history of chronic hepatitis C and a recently diagnosed hepatocellular carcinoma was referred to interventional radiology by his transplant hepatologist for consideration for TIPS to manage repeated bleeding gastroesophageal varices that occurred despite optimal medical and endoscopic management in the setting of decompensated cirrhosis [3]. Previous abdominal magnetic resonance imaging (MRI), computed tomography, and ultrasound exams had demonstrated hepatosplenomegaly with caudate hypertrophy, diffuse coarse nodularity of the liver surface, ascites, recanalization of the umbilical vein, and a lesion meeting imaging diagnostic criteria for hepatocellular carcinoma. However, venous outflow obstruction had not been demonstrated on any study including an MRI with a 1.5 Tesla MAGNETOM Avanto scanner (Siemens, Malvern, PA) and 3 mm slice thickness done 1 month prior to his referral to interventional radiology. His medications included ledipasvir and sofosbuvir for hepatitis C, amiloride and furosemide for ascites, and lactulose for mild hepatic encephalopathy. Laboratory testing on the day of his office visit showed pancytopenia (leukocyte count = 2.7 × 10^3^/*μ*L, hemoglobin = 9.9 g/dL, and platelets = 129 × 10^3^/*μ*L), hypoalbuminemia (2.7 g/dL), and mildly elevated liver enzymes (aspartate transaminase = 56 IU/L and alanine transaminase = 35 IU/L) but normal sodium, bilirubin, and creatinine. Model for End-Stage Liver Disease score was 10 and we therefore chose to proceed with TIPS [4].

**Figure 1:**
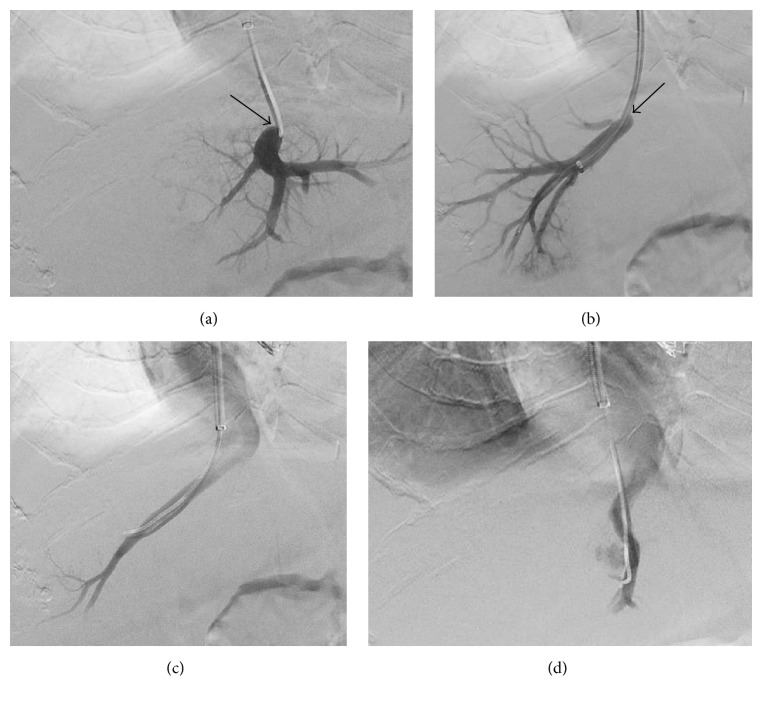
Middle (a) and right (b) hepatic venography showed tight segment stenoses (arrows). Angioplasty was first performed on the right hepatic vein, and repeat venography showed rapid contrast flow into the right atrium (c). The procedure was repeated in the middle hepatic vein (d).

Under fluoroscopy, the middle hepatic vein was accessed and venography demonstrated persistence of contrast in it with minimal flow into the right atrium due to a short segment tight stenosis at the hepatic venous confluence with the IVC due to a venous web not associated with the patient's hepatocellular carcinoma (Figure 1(a)). Pressure gradient measurement across the stenotic portion of the middle hepatic vein was found to be 14 mm mercury greater than the IVC. The right hepatic vein was then accessed and a venogram again demonstrated a short segment tight stenosis at the hepatic vein confluence with the IVC due to a venous web (Figure 1(b)). The procedure was converted to angioplasty as TIPS would not bypass the stenoses near the hepatic vein ostia. A 12 mm × 3 cm Conquest percutaneous transluminal angioplasty dilatation catheter (BARD Peripheral Vascular, Tempe, AZ) was advanced and inflated at the confluence of the right hepatic vein with the IVC. Repeat venography showed rapid contrast flow into the right atrium (Figure 1(c)). Balloon angioplasty was then performed at the confluence of the middle hepatic vein with the IVC as previously mentioned. Repeat venography showed resolution of the stenosis (Figure 1(d)) and repeat pressure measurement demonstrated a normal pressure gradient between the middle hepatic vein and IVC (2 mm mercury), which obviated the indication for TIPS.

At a follow-up appointment 7 weeks after angioplasty, the patient had no ascites or peripheral edema, and his furosemide dose had been reduced from 80 mg to 40 mg daily. He had no further gastrointestinal bleeding at month 7 of follow-up.

**Figure 2:**
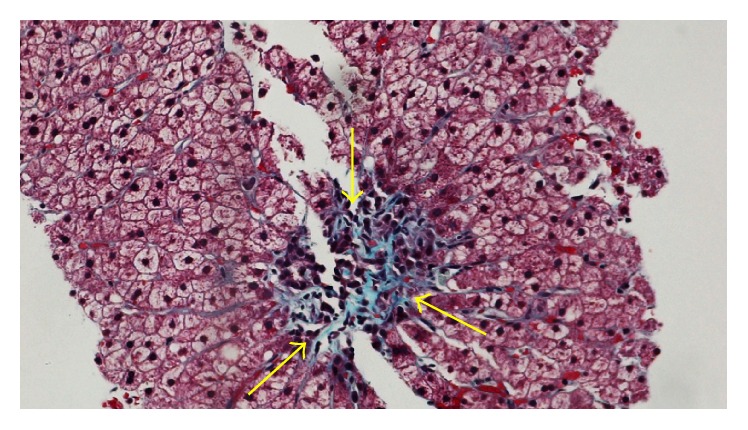
Trichrome staining revealed centrilobular fibrosis (arrows).

Follow-up venography of both the portal and hepatic veins 5 months after angioplasty showed patency of the portal venous system. In the same setting, a liver biopsy was performed in order to clarify the underlying etiology of the disease. Pathologic analysis showed mild chronic portal inflammation and centrilobular fibrosis on a trichrome stain (Figure 2). This finding indicated venous occlusion rather than viral hepatitis which was the cause of cirrhosis.

## 3. Discussion

Budd-Chiari syndrome (BCS) is more common in females, most commonly presents in the 3rd decade of life [5], and can be overlooked in atypical cases. Prior to diagnosis by venography, there had been no suspicion of BCS in this 67-year-old male patient. Importantly, multiple abdominal MRI, computed tomography, and ultrasound exams were performed over the years leading up to diagnosis and treatment without raising suspicion for BCS.

This patient initially presented with, among other signs and symptoms of advanced liver disease, classic BCS findings of abdominal pain and portal hypertension [6]. However, these findings are nonspecific. Other BCS signs and symptoms are also common with advanced liver disease, including vomiting, leg edema, encephalopathy, bleeding portal-systemic varices, jaundice, and fatigue [6].

Many nonspecific imaging findings of BCS, including ascites, caudate lobe hypertrophy, splenomegaly, and irregularities of liver contour [7], were seen in this patient but attributed to cirrhosis secondary hepatitis C, underscoring the difficulty of differentiating BCS from other pathologies that cause cirrhosis. Doppler ultrasonography, which has sensitivity and specificity both >85% [8], is typically used when there is clinical suspicion of BCS. However, technicians must be notified of a high suspicion of hepatic vein pathology to adequately assess this difficult region during abdominal ultrasound.

Current guidelines recommend considering BCS in patients with liver disease after exclusion of other common causes [1]. This, though, ignores the presence of patients with BCS and a coincident common liver pathology such as viral hepatitis. In an era where gastroenterologists and hepatologists are moving towards noninvasive methods of diagnosis and staging of chronic liver disease, patients with multiple insults to the liver may not be properly diagnosed. For instance, nearly 20% of patients with viral hepatitis have been found to have nonalcoholic fatty liver disease after biopsy [9]. Based on the lessons learned from this case, we advise clinicians managing patients with chronic liver disease to ensure a secure diagnosis of the cause of cirrhosis and subsequent decompensation. This requires thorough work up to the time of diagnosis but also careful consideration of secondary contributions from rare second causes of portal hypertension when treating a patient with difficult to manage portal hypertension.
